# Outcomes of Pediatric Traumatic Cardiac Arrest: A 15-year Retrospective Study in a Tertiary Center in Saudi Arabia

**DOI:** 10.7759/cureus.39598

**Published:** 2023-05-28

**Authors:** Mohammed K Almutairi, Muhannad Q Alqirnas, Abdullah Mohammed Altwim, Moustafa S Alhamadh, Munira Alkhashan, Nouf Aljahdali, Bayan Albdah

**Affiliations:** 1 Department of Emergency Medicine, King Abdullah Specialized Children Hospital, Riyadh, SAU; 2 College of Medicine, King Saud Bin Abdulaziz University for Health Sciences, Riyadh, SAU; 3 Department of Emergency Medicine, King Abdulaziz Medical City Riyadh, Riyadh, SAU; 4 Section of Biostatistics, Department of Biostatistics and Bioinformatics, King Abdullah International Medical Research Center, Riyadh, SAU

**Keywords:** motor vehicle accident, major trauma, saudi arabia, pediatrics emergency, pediatric traumatology, traumatic cardiac arrest

## Abstract

Background/objective: Traumatic cardiac arrest (TCA) is the cessation of cardiac pumping activity secondary to blunt or penetrating trauma. The aim of this study is to identify the outcomes of traumatic cardiac arrest in pediatric patients within the local community and report the causes and resuscitation management for the defined cases.

Methods: This was a retrospectively conducted cohort study that took place in King Abdulaziz Medical City (KAMC) and King Abdullah Specialized Children Hospital (KASCH) from 2005 to 2021, Riyadh, Kingdom of Saudi Arabia. The study population involved pediatric patients aged 14 years or less who were admitted to our Emergency Department (ED) and had a traumatic cardiac arrest in the ED.

Results: There were 26,510 trauma patients, and only 56 were eligible for inclusion. More than half (60.71%, n= 34) of the patients were males. Patients aged four years or less constituted 51.79% (n= 29) of the included cases. The majority of patients were Saudis (89.29%, n= 50). The majority of the patients had cardiac arrest prior to ED admission (78.57%, n= 44). The majority (89.29%, n= 50) had a GCS of 3 at ED arrival. The most frequently observed first cardiac arrest rhythm was asystole, followed by pulseless electrical activity and ventricular fibrillation, accounting for 74.55%, 23.64%, and 1.82%, respectively.

Conclusion: Pediatric TCA is high acuity. Children who experience TCA have dreadful outcomes, and survivors can suffer serious neurological impairments. We provided the experience of one of the largest trauma centers in Saudi Arabia to standardize the approach for managing TCA and, hopefully, improve its outcomes.

## Introduction

Trauma is considered the leading cause of morbidity and mortality in children, with traumatic brain injury being the prominent cause [1]. Approximately two to eight children experience pediatric out-of-hospital cardiac arrest (OHCA) for every 100,000 people, with a survival rate of between 6-27% [2-3]. Traumatic cardiac arrest (TCA) is the cessation of cardiac’ pumping activity secondary to a blunt or penetrating trauma [4]. Medical cardiac arrest secondary to an underlying pathological etiology is entirely different on a pathophysiological level from TCA. To clarify, pulseless electrical activity (PEA) is TCA's most frequently encountered initial rhythm. It represents a state of low cardiac output rather than the absence of cardiac output, as in medical cardiac arrest, during which the heart is beating without an appreciable pulse [5]. TCA outcomes are extremely poor, particularly among pediatric and infant age groups, making the value of resuscitation questionable [6,7].

Unlike adults with TCA, preliminary data concerning the survival of pediatric TCA have not shown a notable improvement over time. Moreover, among the pediatric age group, the sequence of events leading to death is not well-defined and studied [8]. Furthermore, consensus regarding the definition of pediatric age group has not been reached yet, rendering the reported incidence and survival rates inaccurate. This notably impacts the reported incidence as well as survival rates, as younger children might be more labile to more severe and lethal injuries [9,10].

To emphasize, the reported survival rate of pediatric TCA ranges from 0-25% in the first 30 days based on the geographical area and the definition of the pediatric age group [11]. To further add to the ambiguity, multiple meta-analyses have found a stark difference in the incidence of pediatric TCA, ranging from 2-20 per 100,000 per year [12]. For all reasons stated, we sought to quantitatively study and categorize pediatric TCA in our local environment over a 15-year timespan and get a better outlook on the incidence, survival rate, causes and management offered to such devastating cases.

## Materials and methods

Design, patients, and setting

This was a retrospectively conducted cohort study that took place in King Abdulaziz Medical City (KAMC) from 2005-2015 and in King Abdullah Specialized Children Hospital (KASCH) from 2015-2021, Riyadh, Kingdom of Saudi Arabia. The study population was pediatric patients aged 14 years or less who were admitted to our Emergency Department (ED) and had a traumatic cardiac arrest in the ED. Data from 2005 to 2015 were obtained via screening of all trauma paper files, and only patients with traumatic cardiac arrest were included. Data from 2016 to 2021 was obtained by screening all cardiac arrest patients' electronic records, and only traumatic cases were included. All non-traumatic cardiac arrest cases and patients older than 14 years were excluded. We also included drowning patients as this is considered traumatic [13].

Data collection

The team gathered data on patients' demographics, arrival times and dates, time until arrival to the hospital, mode of arrival, arrest rhythms, and time of the return of spontaneous circulation (ROSC), Glasgow Coma Scale (GCS) on arrival, mode and mechanism of injury, and injured parts of the body. Also, the number of epinephrine doses, defibrillation, blood products, and intubation during the code blue protocol were collected. Moreover, the outcomes of the patients during discharge, length of ICU and hospital stay, and index of severity of the trauma were collected.

Statistical analysis

Statistical Analysis System (SAS) v. 9.4 (SAS Institute, Cary, NC) was used for data analysis. Categorical variables were presented as frequencies and percentages, whereas numerical variables were presented as mean ± standard deviation. Due to the small sample size, Fisher's exact test was used to test the association between categorical variables. A test was considered significant if the p-value <0.05.

Ethical considerations

The data were collected after obtaining IRB approval from King Abdullah International Medical Research Center (NRC21R/411/10). Access to the data was limited to our research members. The confidentiality of all patients and their families were protected, and no names nor medical record numbers were used. Subjects' privacy and confidentiality were assured, no identifiers were collected, and all data was kept in a secure place within the National Guard Health Affairs (NGHA) premises, both hard and soft copies.

## Results

There were 26,510 trauma patients, only 56 of whom were eligible for inclusion. More than half (60.71%, n = 34) of the patients were males. Patients aged four years or less constituted 51.79% (n = 29) of the included cases. The majority of patients were Saudis (89.29%, n = 50). Most of the cases had cardiac arrest prior to emergency admission (78.57%, n = 44), with almost a third dying at the scene. More than half (64.29%, n = 36) were declared dead in the ED, and less than a third (12.5%, n =7) were alive 30 days following ED admission. Around three-quarters (69.64%, n = 39) of the patients arrived at the hospital via emergency medical services personnel. The majority (89.29%, n = 50) had a GCS of 3 at emergency admission. The most frequently observed first cardiac arrest rhythm was asystole, followed by PEA and ventricular fibrillation, accounting for 74.55%, 23.64%, and 1.82%, respectively. Regarding the second cardiac arrest rhythm, PEA constituted three-quarters of the observed rhythms, and it was the only rhythm in patients with third cardiac arrest (Table 1). 

**Table 1:** Baseline and Demographic Characteristics in Relation to Outcomes of Resuscitations

Baseline and Demographic Characteristics	N	%
Age Group		
≤4 years	29	51.79
>4 years	27	48.21
Gender		
Male	34	60.71
Female	22	39.29
Nationality		
Saudi	50	89.29
Non-Saudi	6	10.71
Location of Cardiac Arrest		
Pre-Emergency Department Admission	44	78.57
After Emergency Department Admission	12	21.43
Mode of Arrival		
By the Emergency Medical Services	39	69.64
By the Family	17	30.36
First Cardiac Arrest Rhythm		
Asystole	41	74.55
Pulseless Electrical Activity	13	23.64
Ventricular Fibrillation	1	1.82
Second Cardiac Arrest Rhythm		
Asystole	2	25.00
Pulseless Electrical Activity	6	75.00
Third Cardiac Arrest Rhythm		
Pulseless Electrical Activity	1	100
Glasgow Coma Scale at Admission:		
3	50	89.29
>3	6	10.71
Hospital Length of Stay		
One Day	37	67.27
>1 Day	18	32.73
Survival at 30 Days		
Yes	7	12.50
Announced Dead in The Emergency Department		
Yes	43	76.79

**Figure 1:**
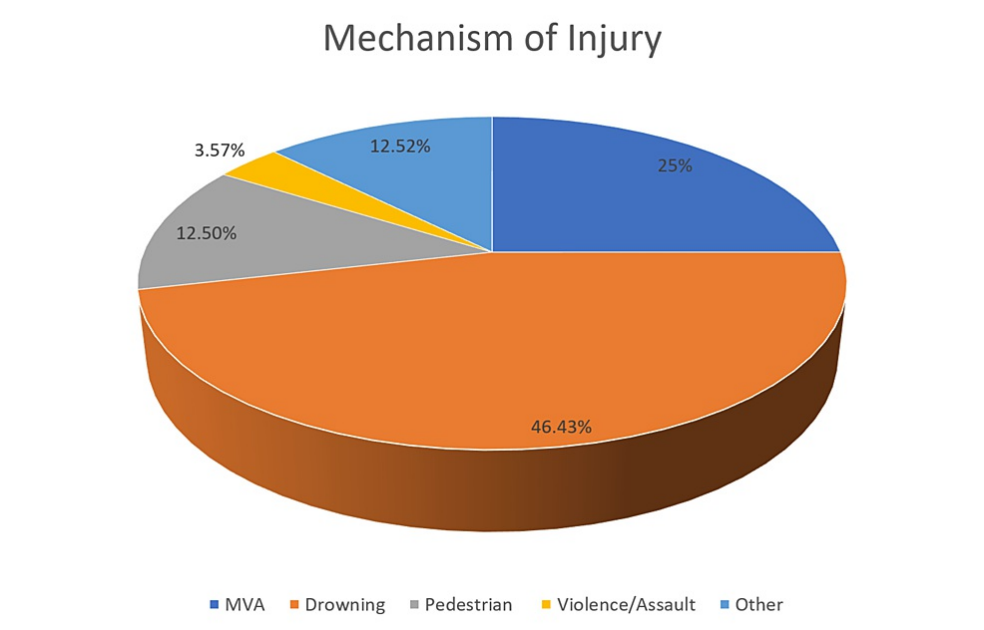
Mechanism of Injury "Other" includes stabbing, burn, electrical injury, and fall MVA: motor vehicle accident

Blunt trauma was responsible for 42.86%, and penetrating trauma was only responsible for 8.93% of TCAs. A quarter (25%, n = 14) were victims of motor vehicle accidents, 10.71% and 7.14% of whom were ejected from or rolled over by a car, respectively. Almost half (46.43%, n = 26) of the patients had experienced drowning (Figure 1). 

**Figure 2:**
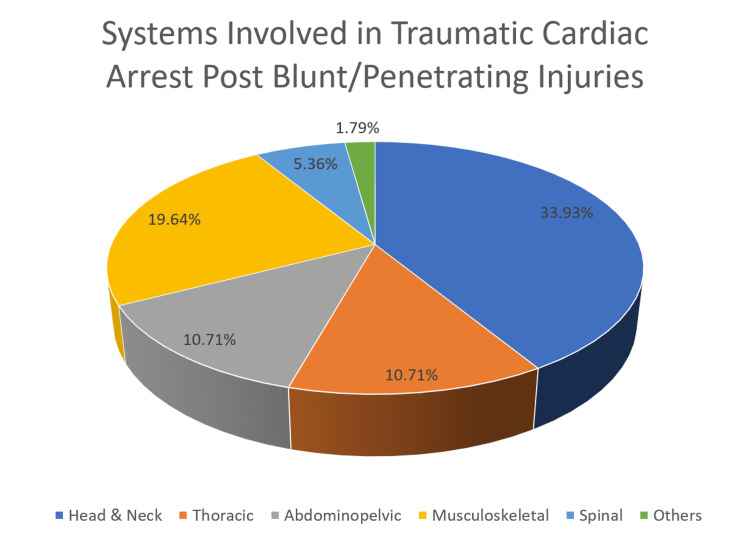
Systems Involved in Traumatic Cardiac Arrest Post Blunt/Penetrating Injuries Other includes: Burn Injury

Almost all had associated injuries with head and neck being the commonest (33.93%. n = 19), followed by musculoskeletal (19.64%, n = 11), thoracic, and abdominopelvic (10.71%, n = 6 for each) injuries (Table 2, Figure 2). Only a few (10.71%, n = 6) cases were suspicious of abuse or intentional injuries. 

**Table 2:** Modes, Mechanisms, and Associated Injuries of Traumatic Cardiac Arrest MVA: motor vehicle accident

Modes, Mechanisms, and Associated Injuries	N	%
Mode of Injury		
Blunt	24	42.86
Penetrating	5	8.93
Other	27	48.21
Mechanism of Injury		
MVA	14	25.00
Pedestrian	7	12.50
Fall	1	1.79
Violence/Assault/Stab	3	5.35
Drowning	26	46.43
Electrical shock/Burn	2	3.57
Others	3	5.36
Associated Injuries		
Head & Neck	19	33.93
Thoracic	6	10.71
Abdominopelvic	6	10.71
Musculoskeletal	11	19.64
Spinal	3	5.36
Intent of Injury		
Non-Intentional	50	89.29
Intentional	6	10.71

Blood products were given for 23.21% (n = 13), slightly more than half (51.79%, n = 29) received >3 epinephrine doses, and only 7.14% (n = 4) needed a defibrillator. In patients who did not die at the scene, 58.93% (n = 33) were intubated with more than half (51.52%) intubated for more than a day (Table 3). 

**Table 3:** In-Hospital Interventions and Related Information in Relation With Outcome of Resuscitations ICU: intensive care unit.

In-Hospital Interventions and Related Information	N	%
Interventions		
Blood Products	13	23.21
Defibrillation	4	7.14
Intubation	33	58.93
Duration of Intubation		
One Day	16	48.48
>1 Day	17	51.52
Number of Epinephrine Doses:		
0-3 Doses	27	48.21
4-10 Doses	29	51.79
ICU Length of Stay		
1-3 Days	8	36.36
4-6 Days	7	31.82
>6 Days	7	31.82

Almost three-quarters (71.14%, n = 5) of the seven patients who survived more than 30 days were ≤4 years old. All of them had cardiac arrest before ED admission, and almost all (85.71%, n = 6) were brought to the hospital by the emergency medical services personnel. Asystole was the most commonly observed first cardiac arrest rhythm, with a percentage of 85.71% (n = 6). Blunt trauma was the most common (28.57%, n = 2) mode of injury while drowning was the most common (57.14%, n = 4) mechanism of injury among the seven survivors (Table 4). 

**Table 4:** Description of the Patients Who Survived ≥30 Days After TCA TCA: traumatic cardiac arrest

Description of the Patients	N	%
Age Group		
≤4 years	5	71.14
>4 years	2	24.57
Gender		
Male	5	71.14
Female	2	24.57
Nationality		
Saudi	7	100
Non-Saudi	0	0
Location of Cardiac Arrest		
Pre-Emergency Department Admission	7	100
After Emergency Department Admission	0	0
Mode of Arrival		
By the Emergency Medical Services	6	85.71
By the Family	1	14.28
First Cardiac Arrest Rhythm		
Asystole	6	85.71
Pulseless Electrical Activity	0	0
Ventricular Fibrillation	1	14.28
Glasgow Coma Scale at Admission		
3	5	71.14
>3	2	24.57
Mode of Injury		
Blunt	2	28.57
Penetrating	1	14.28
Other	4	57.14
Mechanism of Injury		
Pedestrian	1	14.28
Violence/Assault/Stab	2	28.57
Drowning	4	57.14
Head & Neck Injuries		
Yes	2	28.57
Thoracic Injuries		
Yes	1	14.28
Musculoskeletal Injuries		
Yes	1	14.28
Intent of Injury		
Non-Intentional	6	85.71
Intentional	1	14.28

## Discussion

Cardiac arrest is defined as a sudden cessation of cardiac’ pumping activity, and it can be classified based on the underlying cause of traumatic or medical arrest. As the name implies, TCA is a cardiac arrest secondary to blunt or penetrating trauma. Blunt and penetrating trauma needs to be approached in separate ways, necessitating two different treatment algorithms. According to previously published algorithms, a non-traumatic cardiac arrest algorithm includes the inability to feel pain (coma), apnea or gasping breathing, lack of circulation, and pallor or profound cyanosis. TCA algorithm should be initiated when a palpable pulse cannot be felt or a cardiac activity cannot be detected in ultrasonography, which is in line with the most recent International Liaison Committee on Resuscitation (ILCOR) recommendations [14]. Particularly when it has a treatable cause, TCA may occasionally indicate a shallow output state rather than an actual cardiac arrest [15]. Therefore, TCA should be handled differently from medical cardiac arrest.

Although traumatic OHCA [16], which is defined as a stoppage of the heart’s mechanical effort outside of the hospital setting, has a greater survival rate than non-traumatic cardiac arrest, the proportion of patients who survive to be discharged from the hospital is still low [12,17]. Even with the improvement in trauma care and treatment protocols, the survival of cardiac arrest has not significantly changed [18]. The best way to understand modifiable factors of survival and perhaps direct efforts to enhance TCA outcomes is to look into which care setting has the highest survival rates. However, there is an extreme lack of studies in this area on a national level. To the best of our knowledge, this study might be the first to address TCA and try to understand, and hopefully, improve the outcomes [19].

Unexpectedly, children who suffered blunt trauma tend to have favorable outcomes with no obvious reason [20,21]. The literature identifies respiratory impairment as the most frequent cause of cardiac arrest in children [22]. This might partially explain the high survival rate in children who had suffered blunt trauma [23]. A child who experienced cardiopulmonary arrest in a prehospital setting may differ from an adult in a similar setting. To clarify, poor outcomes are frequently linked to hypovolemic cardiac arrest, and conventional CPR is unlikely to stop the high likelihood of bleeding connected to hypovolemic arrest in the prehospital stage. In contrast, providing enough oxygenation may restore spontaneous circulation when apnea precedes cardiac arrest [17].

The treatment of traumatic cardiac arrest is highly organized [24]. Higher-level trauma center accreditation is given to centers with the resources, staff, and experience with severe trauma. It is linked to better results in both adults and children [25]. These resources may be more essential than improved expertise in treating pediatric patients and influencing results [26]. This is consistent with our data showing that higher trauma center treatments are associated with better survival rates.

Quick volume replacement with warm fluids, preferably blood, oxygenation, and ventilatory support should all be given to enhance survival. Therefore, the current treatment algorithms focus on the correction of hypoxia and hypovolemia [27,28]. If not already done in the prehospital setting, applying an appropriately sized pelvic binder is part of the management of non-compressible hemorrhage and reversing hypovolemia for lower girdle injuries in the context of blunt trauma [29]. In our study, blood products were given to almost a quarter of the patients. Slightly more than half (51.79%) received >3 epinephrine doses, and only 7.14% needed a defibrillator. Of the patients who did not experience cardiac arrest at the scene, 58.93% were intubated, with more than half (51.52%) intubated for more than a day.

Pediatric hospitals used resuscitative extracorporeal membrane oxygenation (ECMO) more frequently than mechanical ventilation, which may be related to higher survival but may not entirely account for the survival difference [30,31]. Lower CPR rates in pediatric patients in the hospital may indicate adequate resuscitation before arrival; however, coding variations are also plausible.

In literature, it is agreed by many doctors believe that thoracotomy should be used in cases of penetrating injuries, but they oppose needle pericardiocentesis. There has been a general shift from pericardiocentesis during the past two decades, and there is some indication that subxiphoid pericardiotomy is a more efficient strategy to decompress adult hemopericardium [32]. However, the possibility of clots in the pericardial sac has led many to favor thoracotomy in the context of TCA [33]. This strategy has been supported in previous work for an adult [34], and now experts believe it is suitable for pediatric patients who have experienced penetrating trauma. No consensus could be achieved regarding the decision to do a thoracotomy in cases of forceful trauma to reduce proximal hemorrhage. However, we note that the predominant method of chest decompression taught in pediatric life support courses is still needle thoracocentesis. Patients may choose to perform needle thoracocentesis instead of a thoracostomy [35].

Knowing when to stop CPR is one of the biggest hurdles in any pediatric cardiac arrest. Clinical decision-makers make this choice when the return of spontaneous circulation (ROSC) is highly likely to result in significant neurological disability, even when survival is quite impossible. In these situations, it is crucial to strike a balance between reversing potential causes of arrest while collaborating with the patient's family to provide the best care possible and futility or unfavorable outcomes. A recent comprehensive study of pediatric cardiac arrest did not determine the length of time beyond which resuscitation attempts would be useless or cause severe neurological impairment [36].

It is still unclear when a cardiorespiratory arrest resuscitation effort should stop. This question can only be fully answered by collecting prospective, individualized data on national and international pediatric cardiac arrest registries. The choice to stop resuscitation until this is available will still be made by the resuscitation team and leader based on their assessment of the nature and severity of the injuries, the conclusion of all interventions, and the persistence of the lack of any signs of life.

The most current ILCOR update also discusses the paucity of data supporting intra-arrest prognostic variables for all causes of pediatric cardiac arrest [37]. While we believe doctors managing pediatric TCA can utilize the statement "a lack of response to any therapeutic or invasive treatment is beneficial in identifying failure," other assumptions will need greater clarity using patient-centered outcomes to guide research design.

The study's shortcomings originate from the fact that pediatric TCA continues to be a rare and challenging circumstance that is sometimes not reported or appropriately coded, despite the development of contemporary trauma systems and major trauma centers. In civilian healthcare, most practitioners will probably encounter several cases of TCA incidents involving children. As a result, there is a risk that participants could give in to their collective uncertainty. However, by identifying key stakeholder groups and using this method to contact experts, we have minimized this risk and ensured everyone is included.

## Conclusions

Pediatric TCA is an infrequent but serious condition in Saudi Arabia. Children who experience TCA generally have dreadful outcomes, and survivors suffer from poor neurological outcomes. A subset of individuals who might benefit from intensive resuscitation could not be isolated, which might apply to other countries with comparable healthcare systems. This study shares broad expert agreement on numerous crucial aspects of diagnosing and treating children with TCA. More effort is needed in this area to create an evidence-based therapeutic algorithm.
